# Impact of a Family Clinic Day intervention on paediatric and adolescent appointment adherence and retention in antiretroviral therapy: A cluster randomized controlled trial in Uganda

**DOI:** 10.1371/journal.pone.0192068

**Published:** 2018-03-09

**Authors:** Justin C. Graves, Peter Elyanu, Christine J. Schellack, Barbara Asire, Margaret L. Prust, Marta R. Prescott, Esther Mirembe, Ivan Lukabwe, Betty Mirembe, Joshua Musinguzi, Sarah A. Moberley

**Affiliations:** 1 Applied Analytics Team, Clinton Health Access Initiative, Boston, Massachusetts, United States of America; 2 STD/AIDS Control Program, Ministry of Health, Kampala, Uganda; 3 Clinton Health Access Initiative, Kampala, Uganda; The Ohio State University, UNITED STATES

## Abstract

**Background:**

In 2013, Uganda adopted a test-and-treat policy for HIV patients 15 years or younger. Low retention rates among paediatric and adolescent antiretroviral therapy (ART) initiates could severely limit the impact of this new policy. This evaluation tested the impact of a differentiated care model called Family Clinic Day (FCD), a family-centered appointment scheduling and health education intervention on patient retention and adherence to monthly appointment scheduling.

**Methods:**

We conducted a cluster randomized controlled trial, from October 2014 to March 2015. Forty-six facilities were stratified by implementing partner and facility type and randomly assigned to the control or intervention arm. Primary outcomes included the proportion of patients retained in care at 6 months and the proportion adherent to their appointment schedule at last study period scheduled visit. Data collection occurred retrospectively in May 2015. Six patient focus group discussions and 17 health workers interviews were conducted to understand perspectives on FCD successes and challenges.

**Results:**

A total of 4,715 paediatric and adolescent patient records were collected, of which 2,679 (n = 1,319 from 23 control facilities and 1,360 from 23 intervention facilities) were eligible for inclusion. The FCD did not improve retention (aOR 1.11; 90% CI 0.63–1.97, p = 0.75), but was associated with improved adherence to last appointment schedule (aOR 1.64; 90% CI 1.27–2.11, p<0.001). Qualitative findings suggested that FCD patients benefited from health education and increased psychosocial support.

**Conclusion:**

FCD scale-up in Uganda may be an effective differentiated care model to ensure patient adherence to ART clinic appointment schedules, a key aspect necessary for viral load suppression. Patient health outcomes may also benefit following an increase in knowledge based on health education, and peer support. Broad challenges facing ART clinics, such as under-staffing and poor filing systems, should be addressed in order to improve patient care.

## Introduction

In Uganda, the adoption of a test-and-treat policy for HIV patients 15 years or younger in late 2013 resulted in the rapid expansion of the ART eligible paediatric and adolescent population. With approximately 21,000 patients already active in pre-ART care, these patients became eligible for immediate initiation onto ART.[1] However, in 2013 the 12 months ART retention rate among children was about 78.7%.[1, 2] The Ministry of Health (MOH) was concerned about the potential for such a large number of the newly initiated paediatric and adolescent patients to be lost to care with deleterious effects on their health and survival.

Younger patients often depend on their caregivers to bring them to the facility for their appointments and to pick up medicine. However, caregiver attendance at the ART clinic is associated with loss of income based on time away from income-earning activities and additional costs due to transport. When caregivers are also HIV positive, their appointments may not be scheduled on the same day, leading to increased costs or difficult choices. Such burdens may present a challenge for families to remain adherent to appointments and their ART treatment regime.

Families also may face challenges related to the disclosure of their child’s HIV status and lack of confidence in explaining the disease to their child.[3] A recent study in Uganda indicated that only 56% of HIV-positive children were aware of their HIV status.[4] Evidence suggests that adolescents who do not know their status are significantly less likely to be adherent to ART.[5]Likewise studies in Uganda and Tanzania have shown that having HIV status disclosed by caregivers was associated with good treatment adherence, along with residing with a biological parent and having a supportive relationship with a parents.[6, 7] HIV awareness and education becomes particularly important for adolescents who may be becoming sexually active however basic knowledge of HIV and how it is spread remains very low in adolescents,[8] and well below the target coverage of 95%.[9]

Evidence supporting the effectiveness of a family-centered approach to paediatric and adolescent ART care is limited due to small sample sizes and non-rigorous methods. Some ART facilities in Uganda already provide a model of family-centered care,[10] and evaluations of this approach have shown positive results, including a dramatic 43-fold increase in children enrolled in care and a 23-fold increase in children on ART over a 7-year period after the introduction of the family care model.[11] Additionally, an intervention using an adolescent peer support group was also found to be an effective way to improve adherence to ART and improve self-image, with previously fearful adolescents perceived to become more confident and outgoing.[12]

In order to improve retention rates and adherence to ART care among children and adolescents in view of maximizing the benefit of Uganda’s new ART guidelines, the MOH designed an intervention called the Family Clinic Day (FCD), to be pilot tested before potentially scaling-up nationally. The primary objective of this study was to determine whether the FCD intervention was able to improve the proportion of paediatric and adolescent patients that were retained in care and adherent to their treatment schedule.

## Methods

### Study design

We conducted a clustered, stratified, two-arm parallel group (1:1), randomized controlled trial. The study was conducted at government ART-accredited health center level III facilities (HCIIIs), health center level IV facilities (HCIVs), and general hospitals (GHs) across the Central 1, Central 2, Eastern and Northern regions of Uganda. Facility-level clusters were included if they were supported by an implementing partner and had 30 or more children under the age of 15 years who were active in pre-ART or ART care at the start of the intervention, in order to ensure a sufficiently large patient population to justify usage of the FCD in those selected sites.

Facility clusters were excluded based on implementing partners, if there was an insufficient total number of ART-enrolled patients at the facility, or if already implementing a program similar to the FCD intervention.

Stratification of facility-level clusters was selected on the basis of implementing partner (Mildmay, NUHITES and StarE implementing partners) and facility type (health center III, health center IV, hospital levels) and randomly assigned to the control arm or the intervention arm. All HIV-infected children on ART seeking care among intervention facilities, including their immediate family members, were eligible to receive the FCD intervention as implemented by trained facility healthcare workers. All HIV-infected patients among control facilities received ART standard of care.

The study lasted for 6 months at all facilities, beginning on 1 October 2014 and ending on 31 March 2015. Prior to the official study start during the month of September 2014, support was provided to prepare the FCD schedules and plans among all randomly selected intervention facilities. All ART care was provided following standard MOH guidelines.

This study received approval from the MOH in Uganda and from the MildMay Uganda Research and Ethics Committee and the Uganda National Council for Science and Technology, and additionally was registered with the Pan African Clinical Trial Registry (PACTR201508001149239).

### FCD intervention

FCD was designed as a differentiated care model to ensure prioritization of HIV treatment and counselling for any paediatric and adolescent patients, including their immediate family members. Following consultation with experienced counsellors, a set of resources were developed to target the educational needs specific to adolescents and caregivers of paediatric HIV patients. In addition to the intervention package described below, all standards of HIV care were provided to eligible patients when attending the clinic on an FCD. The FCD intervention involved three main components as part of the differentiated care model:

1. **Patient scheduling**: Any age-eligible paediatric and adolescent patient, including their family unit, was scheduled to attend the ART clinic on a regularly occurring designated day in order to re-route all such patient appointments onto the same day. Calendars and reminder cards helped schedule eligible patients to attend their next appointment on a FCD. Promotion of the FCD intervention was strictly facility-based among intervention sites.
2. **Health education:** Two separate specialized health education sessions were conducted during each FCD; one session targeted adolescent patients while the other was conducted with paediatric patient caregivers. Each session was led by an expert client, a long-term adherent patient, using high quality illustrated flip charts specifically aimed at increasing knowledge of HIV basics and background, adherence, disclosure, puberty, sexual and reproductive health, and life skills among other topics. FCD clients were highly encouraged to attend HIV health education sessions during FCDs.
3. **Patient flow:** During a designated FCD, children, adolescents and their families were prioritized for care over other patients. Patients ineligible for FCD were purposefully scheduled on non-FCD designated clinic days.

Clinicians and ART staff among intervention facilities were empowered to determine their respective FCD schedules. Trainers helped guide decision-making, which was based on calendar inputs using pre-existing ART clinic schedules; facilities implementing 2 or fewer ART clinic days per week were encouraged to schedule 1 FCD per month, while all other facilities implemented FCDs on a weekly basis. Factors such as national school holiday schedules, and other clinic-based or local community-based events helped select specific days for FCD implementation.

Created in collaboration with the Ugandan MOH, a standardized FCD implementation guidebook was used as the primary training tool of a two-step cascade-training course. This training was first conducted centrally for master trainers, including senior chief medical officers and senior nursing officers who then stepped down training regionally among all participating facilities. The 2-day training covered all aspects of FCD implementation including study background, FCD introduction, intervention set-up, scheduling, counseling, and record keeping.

### Patient participant eligibility

Study participants included paediatric and adolescent patients attending control or intervention facilities who were aged between 19 months and 19 years, and who were ever active in care during the first three months of the study period from 1 October 2015 to 31 December 2015 and had ART care cards. Children aged less than 19-months were excluded because they received their care at a separate facility. Among intervention facilities, eligible patients and their respective family units automatically received FCD by default as part of a facility-wide MOH-led programmatic rollout of the intervention. Within each facility cluster, paediatric and adolescent patients included in the analysis were then selected using simple random sampling without replacement, based on a sampling interval applied to the total pool of eligible paediatric and adolescents within each study facility.

A secondary adult patient population was also sampled from facilities in order to determine whether FCD had any negative impact on patients who were ineligible for FCD. Therefore, any adults aged over 19 years of age who had an ART care card and were active in care at least once from 1 October 2015 to 31 December 2015 were eligible for inclusion in the secondary analysis. Within the intervention arm, these adult participants fell outside of the FCD family unit and maintained standard of care by visiting the ART clinic on non-FCD days. Like paediatric and adolescent patients, adult study participants were also selected using simple random sampling without replacement based on a sampling interval applied to the total pool of active adults within each respective facility.

All patient-level quantitative data were collected retrospectively by extracting relevant information from ART care cards and ART registers. During data collection, strict measures were implemented to mask all patient identification information such as names, phone numbers and addresses, and the assignment of unique random codes to each participant ensured de-identification. Therefore as agreed with ethical boards and the Ugandan MOH, consent was not provided on an individual level.

Written consent was acquired from all caregiver and healthcare worker participants prior to qualitative interviews conducted in the form of focus group discussions.

### Outcomes

Individual-level primary outcomes included 1) the retention in care of paediatric and adolescent patients, and 2) adherence to appointment schedule of paediatric and adolescent patients. Patients were classified as retained in care if they attended any ART clinic appointment at least once over the last 3 months of the study period, from 1 January 2015 to 31 March 2015. Any patients who transferred out to another facility were excluded. Patients were classified as adherent to appointment schedule if they returned to the clinic for their most recent appointment in a number of days that was equal to or less than the number of ARV pills prescribed at their last appointment. Patients were excluded from the adherence analysis if they had less than two appointment visits during the study period. An effective intervention was based on statistically significant increases in the proportion of each outcome within the FCD intervention arm compared to the control arm.

Secondary outcomes examined whether deleterious effects of the FCD intervention existed among adult patients who were ineligible for the FCD. To evaluate whether this was the case, we specifically conducted a non-inferiority test in this group to ensure that the proportion of adults retained in care did not statistically decrease in the intervention arm compared to control arm Pregnant women were excluded because they receive care at the antenatal clinic rather than the main HIV clinic.

### Data collection and statistical analyses

Quantitative data were collected retrospectively in May 2015 by trained enumerators who extracted data from ART registers and patient ART Care Cards of sampled participants. Our study used an electronic data capture survey tool that had been programmed onto portable android tablets with the SurveyCTO software. Simple random sampling without replacement selected the sample of study participants from the total number of eligible clients listed within each study facility.

The study sample size and power was determined based on child-level retention outcomes, using the below equation by Hayes et al designed for cluster randomized trials [13, 14].

$$c=2+{\left(z_{\frac{\alpha }{2}}+z_{\beta }\right)}^{2}*\frac{\left[\pi_{0}(1-\pi_{0})+\pi_{1}(1-\pi_{1})]*[1+(m-1)\rho \right]}{m{\left(\pi_{0}-\pi_{1}\right)}^{2}}$$

All parameter estimates were informed by a Ugandan MOH dataset assessing retention of paediatric patients at 6 months post ART initiation [15], where the retention outcome at 6 months was estimated at 70% among control sites (π_0_) with an intra-cluster correlation coefficient (rho) value of 0.03. Based on these inputs, the inclusion of 46 total facility-level clusters achieved a minimum detectable effect size of 8 percentage points at 80% power at 90% confidence level (Table 1).

**Table 1:** Sample size equation parameters.

Parameter	Value
Alpha (α)	0.10
Z_α/2_	1.68
Power (β)	0.8
Unadjusted units sampled (*m*)	80
Adjusted units sampled with 50% missing data	40
Proportion in control group (π_0_)	0.70
Proportion in intervention group (π_1_)	0.78
Effect size to detect	0.08
Rho (ρ)	0.03
c (clusters per arm)	23

This calculation was done using anecdotal evidence from ART register reviews based on a conservative average of 50% of the 80 individuals sampled per cluster will have available and complete data, in order to ensure minimum sampling assumptions were feasible to adequately power the study. Therefore, collecting data from a minimum of 40 patients from each of the 46 facilities would ensure sufficient statistical power.

Using an equation that tests for non-inferiority of the post-intervention adult population,[14] and the same rho estimate, the intended sample of 35 patients per facility (allowing for 50% of patients with incomplete or unavailable files) at 80% power and 90% confidence level provided the ability to recognize a 7% difference in retention (delta) among the adult population.

Paediatric and adolescent population estimate outcomes were weighted to account for unequal probability of selection and chi-squared tests were used to detect statistical significance. Secondary outcomes among adult populations were unweighted because the total active adult population was not confirmed by facility.

To further describe the differences between the arms, the relative odds of retention and adherence were examined. Crude and adjusted odds ratios using logistic generalized estimating equation (GEE) regression models were used for the primary outcome comparing the intervention arm to the control arm at the individual-level while accounting for cluster, weighted to account for unequal probability of selection within facility, and characteristics to generalize the strata used for randomization. Adjusted GEE regression models reported for child retention and adherence controlled for ART initiation category, age, region, gender, whether the client was not present during appointment and instead was represented by an immediate adult family member (ART representation) and facility level.

Adult non-inferiority testing was carried out by examining the absolute difference in the mean proportion retained in care between the intervention and control arms. To account for any facility-level clustering and variations in facility population sizes, facility-level proportions of retention were calculated (i.e. those retained over the eligible adult population) and these facility-level proportions were used to create the mean proportions for the intervention and control arms and then compared using a t-test. Our *a priori* threshold of non-inferiority was that the mean proportion retained in the intervention arm could be no more than 0.07 units (or 7%) lower than the mean proportion retained in the control arm. All quantitative data analysis was conducted using Stata version 13.

### Qualitative data

To better understand perceptions and experiences of the FCD intervention, focus group discussions and interviews were conducted in March 2015. Six FCD facilities were purposefully selected to include a range of facility types and regions determined on the basis of theoretical saturation of participant responses [16]. Interviews were conducted with 17 healthcare workers including 5 ART in-charges, 1 doctor, 5 nurses and 6 expert clients. Six focus groups, including 19 individuals, were conducted with adolescent patients aged 14 or older. Groups with adolescents were smaller than expected due to logistical challenges and ranged in size from two to five participants. Three of the focus groups were with male adolescents and 3 were with female adolescents. Six additional focus groups, including 38 individuals, were conducted with caregivers of paediatric and adolescent patients. Groups with caregivers ranged in size from four to eight participants.

Three trained interviewers used semi-structured interview guides to address respondent experiences and perceptions related to the success of FCD scheduling, health education effectiveness and impact, perception of psychosocial benefits, experience with patient flow, and overall feedback on the FCD intervention. Interviews with staff at the facility were typically conducted in English, except for the expert client interviews, which were conducted in the local language. All focus group discussions with patients and caregivers were conducted in the local language.

Discussions were audio recorded, then translated and transcribed in English. Two members of the research team developed a code structure and coded the initial transcripts, using the constant comparative method to systematically code the transcripts. [17, 18] Once the coding structure was established, one researcher applied the codes to the transcripts. Dedoose software was used to facilitate data organization and retrieval.

## Results

**Fig 1:**
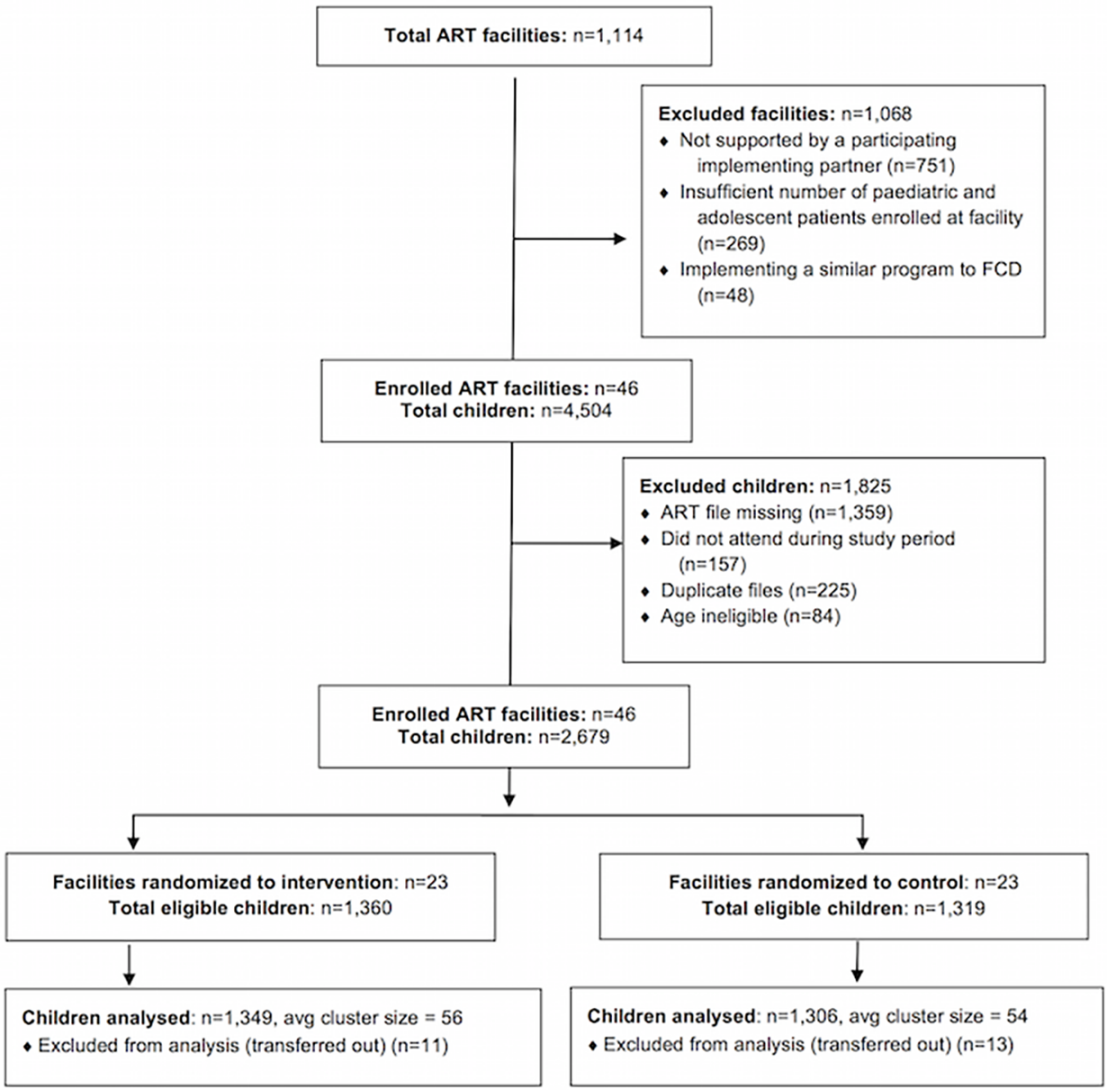
CONSORT study flow chart.

Among 1,114 total government ART facilities in Central 1, Central 2, Eastern and Northern regions of Uganda, 46 facilities were stratified and randomly assigned to the control or intervention arm following exclusion criteria (Fig 1, S1 Table). Among the 23 facilities randomly assigned to the intervention, all HIV-infected children on ART, including their immediate family members or caregivers, were eligible to receive the FCD intervention.

Table 2 outlines the characteristics of the 46 participating facilities; most facilities were either HCIII level (39%) or HCIV level (46%), with fewer hospitals (15%). The majority of participating facilities came from the Northern region (57%), followed by Central region (24%) and Eastern region (20%). These characteristics were balanced across control and intervention arms.

**Table 2:** Facility characteristics by treatment arm.

CHARACTERISTICS	CONTROL (n = 23)	INTERVENTION (n = 23)	P-VALUE	TOTAL
***Facility level***	*** ***	*** ***	***0*.*91***	*** ***
Health Centre III	9 (39.1%)	9 (39.1%)		18 (39.1%)
Health Centre IV	11 (47.8%)	10 (43.5%)		21 (45.4%)
Hospital	3 (13%)	4 (17.4%)		7 (15.2%)
***Region***			***0*.*90***	
Central 1&2	6 (26.1%)	5 (21.7%)		11 (23.9%)
Eastern	4 (17.4%)	5 (21.7%)		9 (19.6%)
Northern	13 (56.5%)	13 (56.5%)		26 (56.5%)

Data presented are n(%)

A total of 4504 paediatric and adolescent patients were identified as eligible for inclusion in the final sample for analysis, of which, 2679 (59%) had patient files that were available and complete (n = 1319 from control facilities and 1360 from intervention facilities). Among all children eligible for inclusion in the analysis (n = 4,504), there was a large amount of missing data (n = 1359) due to lost ART care cards for patients who were otherwise listed as active (Fig 1). The weighted paediatric and adolescent patient sample characteristics were balanced across intervention and control facilities, with no significant differences (Table 3). FCD exposure was high, as among sampled patients in the FCD arm included in the analysis, 80% (1082/1360) had ever attended a scheduled FCD. Furthermore, over half of patients in the FCD arm (685/1360) attended between 75–100% of scheduled FCD in their respective clinic. In the control arm, 100% of sampled patients (n = 1319) were never exposed to the FCD intervention.

**Table 3:** Weighted characteristics of child and adolescent patients by treatment arm.

CHARACTERISTICS	CONTROL (n = 1319)	INTERVENTION (n = 1360)	P-VALUE
***Initiation category***			***0*.*49***
Within past 3 months	137 (6.0%)	166 (7.2%)	
4–11 months	739 (32.6%)	660 (28.7%)	
12–24 months	368 (16.2%)	355 (15.4%)	
More than 24 months	1023 (45.1%)	1121 (48.7%)	
***Age***			***0*.*5***
19–23 months	14 (0.6%)	6 (0.3%)	
2–4 years	408 (17.6%)	419 (17.8%)	
5–9 years	991 (42.8%)	1114 (47.3%)	
10–15 years	647 (27.9%)	581 (24.7%)	
16–19 years	256 (11.1%)	236 (10.0%)	
***Region***			***0*.*69***
Central 1 & 2	630 (26.4%)	350 (14.7%)	
Mid-Eastern	385 (16.1%)	357 (15.0%)	
Mid-Northern	1370 (57.4%)	1669 (70.3%)	
***Gender***			***0*.*74***
Male	972 (43.1%)	908 (42.0%)	
Female	1284 (56.9%)	1256 (58.0%)	
Ever Represented*			***0*.*68***
Yes	1382 (42.0%)	1299 (45.3%)	
No	1002 (58.0%)	1077 (54.7%)	
***Facility Level***			***0*.*63***
Hospital	497 (20.8%)	768 (32.3%)	
Health Center III	1378 (57.8%)	935 (39.3%)	
Health Center IV	510 (21.2%)	673 (28.3%)	

Data presented are n(%).

*Ever represented is a measure of whether a child was represented by a caregiver at any point during the period instead of attending the clinic in person

A total of 3224 records of adult patients were identified as eligible for inclusion in the sample, of which, 2347 (73%) had patient files that were available and complete. Overall, the characteristics were well-balanced among adult patients from intervention and control sites, with no significant differences (Table 4).

**Table 4:** Weighted characteristics of adult patients by treatment arm.

CHARACTERISTICS	CONTROL (n = 1251)	INTERVENTION (n = 1119)	P- VALUE
***Initiation category***			***0*.*47***
Within past 3 months	76 (6.3%)	51 (4.7%)	
4–11 months	253 (21.0%)	183 (16.7%)	
12–24 months	253 (21.0%)	234 (21.4%)	
More than 24 months	624 (51.7%)	625 (57.2%)	
***Age (years)***			***0*.*9***
19	70 (5.8%)	60 (5.5%)	
20–49	921 (76.5%)	842 (77.5%)	
50+	213 (17.7%)	185 (17.0%)	
***Region***			***0*.*72***
Central 1 & 2	351 (28.1%)	257 (23.0%)	
Mid-Eastern	157 (12.6%)	259 (23.2%)	
Mid-Northern	743 (59.4%)	603 (53.9%)	
***Gender***			***0*.*86***
Male	397 (33.7%)	361 (34.1%)	
Female	781 (66.3%)	698 (65.9%)	
Ever Represented*			***0*.*83***
Yes	1002 (80.1%)	887 (79.3%)	
No	249 (19.9%)	232 (20.7%)	
***Facility Level***			***0*.*91***
Hospital	128 (10.2%)	171 (15.3%)	
Health Center III	565 (45.2%)	482 (43.1%)	
Health Center IV	558 (44.6%)	466 (41.6%)	

Data presented are n(%).

*Ever represented is a measure of whether a patient was represented by another person at any point during the period instead of attending the clinic in person.

Overall, 1.0% of the sample at control sites (weighted n = 24) and 0.8% of the sample at intervention sites (weighted n = 21) were excluded due to transferring out of the facility. Overall, a high proportion of paediatric and adolescent patients were retained in ART care at the end of the study period; 91.0% (n = 2148, 90%CI 87.1–93.8) from the control group and 92.0% (n = 2166, 90%CI 87.8–94.8) from the intervention group. Crude GEE regression analysis suggested there was no significant difference in the odds of retention between the two study arms (90% CI 0.56 − 1.88, p value 0.94) (Table 5, Model 1).

**Table 5:** Models 1–4 − weighted generalized estimation equation regression analysis of adolescent and pediatric patient retention and adherence.

CHARACTERISTICS	MODEL 1 RETENTION		MODEL 2 RETENTION		MODEL 3 ADHERENCE		MODEL 4 ADHERENCE	
	*cOR*	*90% CI; p value*	*aOR*	*90% CI; p value*	*cOR*	*90% CI; p value*	*aOR*	*90% CI; p value*
***Treatment Arm***								
Control	ref	-	ref	-	ref	-	ref	-
Intervention	1.03	0.56 − 1.88; 0.94	1.11	0.63 − 1.97; 0.75	1.57	1.21 − 2.01; <0.01	1.67	1.27 − 2.11; <0.01
***Initiation category***								
Within past 3 months	-	-	ref	-	-	-	ref	-
4–11 months	-	-	0.22	0.05 − 0.87; 0.07	-	-	0.83	0.50 − 1.36; 0.53
12–24 months	-	-	0.22	0.07 − 0.76; 0.05	-	-	0.88	0.52 − 1.50; 0.70
More than 24 months	-	-	0.27	0.09 − 0.85; 0.06	-	-	0.75	0.46 − 1.22; 0.33
***Age***								
19–23 months	-	-	ref	-	-	-	ref	-
2–4 years	-	-	1.52	0.34 − 6.87; 0.65	-	-	1.01	0.32 − 3.15; 0.99
5–9 years	-	-	1.21	0.25 − 5.74; 0.84	-	-	1.13	0.39 − 3.28; 0.85
10–15 years	-	-	0.95	0.21 − 4.31; 0.95	-	-	1.26	0.38 − 4.22; 0.75
16–19 years	-	-	0.68	0.14 − 3.36; 0.70	-	-	0.87	0.29 − 2.61; 0.84
***Region***								
Central 1 & 2	-	-	ref	-	-	-	ref	-
Mid-Eastern	-	-	2.59	1.03 − 6.52; 0.09	-	-	0.96	0.63 − 1.48; 0.89
Mid-Northern	-	-	0.59	0.33 − 1.05; 0.13	-	-	0.72	0.49 − 1.05; 0.15
***Gender***								
Male	-	-	ref	-	-	-	ref	-
Female	-	-	1.14	0.88 − 1.47; 0.40	-	-	1.12	0.89 − 1.42; 0.41
Ever Represented*								
Yes	-	-	ref	-	-	-	ref	-
No	-	-	1.93	1.37 − 2.71; <0.01	-	-	1	0.85 − 1.18; 0.97
***Facility Level***								
Hospital	-	-	ref	-	-	-	ref	-
Health Center III	-	-	1.13	0.43 − 2.97; 0.84	-	-	0.81	0.57 − 1.15; 0.33
Health Center IV	-	-	1.31	0.53 − 3.28; 0.62	-	-	0.99	0.69 − 1.43; 0.98

For all factors, each category is compared to the base category. cOR = crude odds ratio. aOR = adjusted odds ratio. CI = confidence interval. Ref = reference category.

*Ever represented is a measure of whether a child was represented by a caregiver at any point during the period instead of attending the clinic in person

The regression model indicated patients enrolled in care at an intervention facility had an 11% higher odds of being retained in care as compared to that of control facilities (Table 5, Model 2), but the difference was not significant (90% CI 0.63 − 1.97, p value 0.75) when adjusting for ART initiation category, age, region, gender, representation, and facility level.

We conducted sensitivity analyses including defining retention by using the number of days elapsed between the last scheduled follow-up and last visit, if any. Those patients who were between 7–90 days late for their last scheduled follow-up were considered lost but not lost to follow-up. Using these less stringent criteria, the proportion of patients retained in care dropped slightly among the FCD intervention arm (82.3%), but this was still not significantly different from the control arm (84.9%).

The weighted findings indicated 65.5% (90%CI 59.4–71.1) of paediatric and adolescent participants from the intervention facilities were adherent to their appointment schedule compared to 55.3% (90%CI 51.7–58.9) of participants from control facilities (t-test p-value <0.01). The adjusted odds ratio from the weighted GEE regression model indicated patients receiving care from intervention were 67% more likely to be adherent to their appointment that participants from control facilities (90% CI 1.27−2.11, p value <0.001) (Table 5, Model 4).

A total of 2370 adults were included in the analysis for retention, (23 transferred out; of which 13 and 10 were from the intervention and control group respectively). Overall, there was also a high proportion of adults retained in ART care at the end of the study period; 93.4% (90%CI 91.2–95.1) from the control group and 91.9% (90%CI 88.9–94.1) from the intervention group (p = 0.47) (Table 6, Model 1). The adjusted odds ratio for patients receiving care from intervention were 15% less likely to be retained in ART care than participants from control facilities (Table 6), when adjusting for ART initiation category, age, region, gender, representation, and facility level, however this was not significant (90% CI 0.49−1.46, p value 0.62) (Table 6, Model 2).

**Table 6:** Models 1–4 − unweighted generalized estimation equation regression analysis of adult patient retention and adherence.

CHARACTERISTICS	MODEL 1 RETENTION		MODEL 2 RETENTION		MODEL 3 ADHERENCE		MODEL 4 ADHERENCE	
	cOR	90% CI; p value	aOR	90% CI; p value	cOR	90% CI; p value	aOR	90% CI; p value
***Treatment Arm***	*** ***	*** ***	*** ***	*** ***	*** ***	*** ***	*** ***	*** ***
Control	ref	-	ref	-	ref	-	ref	-
Intervention	0.79	0.47 − 1.35; 0.47	0.85	0.49 − 1.46; 0.62	1.31	0.97 − 1.75; 0.14	1.33	1.00 − 1.78; 0.11
***Initiation category***	*** ***	*** ***	*** ***	*** ***	*** ***	*** ***	*** ***	*** ***
Within past 3 months	-	-	ref	-	-	-	ref	-
4–11 months	-	-	0.06	0.01 − 0.21; <0.01	-	-	0.19	0.09 − 0.40; <0.01
12–24 months	-	-	0.06	0.07 − 0.24; <0.01	-	-	0.29	0.14 − 0.61; <0.01
More than 24 months	-	-	0.11	0.03 − 0.39; <0.01	-	-	0.3	0.14 − 0.63; <0.01
***Age (years)***	*** ***	*** ***	*** ***	*** ***	*** ***	*** ***	*** ***	*** ***
<20	-	-	ref	-	-	-	ref	-
20–49	-	-	1.11	0.59 − 2.10; 0.78	-	-	0.94	0.61 − 1.44; 0.81
50+	-	-	1.01	0.52 − 1.96; 0.98	-	-	0.92	0.64 − 1.32; 0.71
***Region***	*** ***	*** ***	*** ***	*** ***	*** ***	*** ***	*** ***	*** ***
Central 1 & 2	-	-	ref	-	-	-	ref	-
Mid-Eastern	-	-	0.62	0.29 − 1.32; 0.30	-	-	1.12	0.66 − 1.91; 0.73
Mid-Northern	-	-	0.45	0.24 − 0.84; 0.04	-	-	0.95	0.64 − 1.42; 0.84
***Gender***	*** ***	*** ***	*** ***	*** ***	*** ***	*** ***	*** ***	*** ***
Male	-	-	ref	-	-	-	ref	-
Female	-	-	1.11	0.88 − 1.41; 0.46	-	-	1.14	0.96 − 1.35; 0.20
Ever Represented*	*** ***	*** ***	*** ***	*** ***	*** ***	*** ***	*** ***	*** ***
Yes	-	-	ref	-	-	-	ref	-
No	-	-	1.81	1.21 − 2.71; 0.02	-	-	0.74	0.57 − 0.96; 0.06
***Facility Level***	*** ***	*** ***	*** ***	*** ***	*** ***	*** ***	*** ***	*** ***
Hospital	-	-	ref	-	-	-	ref	-
Health Center III	-	-	0.8	0.45 − 1.43; 0.53	-	-	1.43	0.84 − 2.43; 0.27
Health Center IV	-	-	0.83	0.45 − 1.54; 0.62	-	-	1.54	0.95 − 2.48; 0.14

For all factors, each category is compared to the base category. cOR = crude odds ratio. aOR = adjusted odds ratio. CI = confidence interval. Ref = reference category.

*Ever represented is a measure of whether a patient was represented by another person at any point during the period instead of attending the clinic in person

The t-test for non-inferiority suggested an absolute difference in the mean proportion retained between the intervention and control arms of -0.02 (90% CI -0.06 to 0.02). As our estimate and confidence interval for mean proportion retained in our intervention arm was not 0.07 units lower than the mean proportion retained in our control arm, we concluded that retention of the adults in the intervention arm was not inferior to that of the control arm.

The unweighted findings indicated 78.6% (90%CI 75.7–81.4) adult participants from the intervention facilities were adherent to their appointment schedule compared to 74.5% (90%CI 69.5–79.0) of participants from control facilities, as the likelihood of adherence did not differ between intervention and control participants (aOR 1.33; 90% CI 1.00 − 1.78, p value 0.11) (Table 6, Model 4), when adjusting for ART initiation category, age, region, representation, and facility level.

In focus group discussions, patients described ways in which the FCD program positively impacted their well-being and clinic experience in these 4 key areas:

1. **Health education:** Participants reported that family-centered topics discussed during health education sessions were relevant to their needs and challenges and presented in an accessible format regardless of educational level. Caregivers and adolescents reported what they remembered and learned most were related to: adherence to medications, preventing child neglect and social care for children, condom use and reproductive health and good nutrition.
2. **Clinic congestion:** Participants reported it takes approximately 3–6 hours for all patients to be seen on FCDs, and that in general, the waiting time was shorter on FCDs compared to the general ART clinic days. This may be related to several factors: prior to FCD, some clinics had no appointment systems so patients were not evenly spread across clinic days; group health education and joint family clinical visits allow health workers to attend to patients more quickly; and in some cases, there were fewer patients on an FCD than on other days based on the facility population demographics.
3. **Appointment and file management:** Many participants described the positive impact of the tools provided as part of the FCD package including folders, appointment management and support to organize patient files. Interviewed patients confirmed their files were more easily found, and lost files appeared to be less frequent. Furthermore, patients described how the appointment reminder cards were helping them adhere to their appointment schedule and come on the correct day.
4. **Family and peer psychosocial support:** Within family groups, participants discussed a number of improvements in the relationships and support networks as a result of the FCD program. First, that the FCD program had facilitated disclosure of HIV status within the family unit. Previously, caregivers were collecting drugs at the facility, but health education about HIV status disclosure to a child had never been discussed so children did not understand the purpose of medication. In some cases, adult members of the same family were collecting drugs from different facilities and had not disclosed status to each other. Thanks to improved disclosure of HIV status in the FCD intervention, family members could remind each other to take medications and adhere to appointment schedules. Also, within families the FCD program may have helped engaging certain types of family members who were not previously involved in care. Specifically, caregivers and healthcare workers reported increased male involvement in seeking ART services and ensuring that the whole family was receiving care. Non-patient caregivers (who are not HIV-positive) more frequently attended the facility in support of their children. Outside the family unit, adolescents and caregivers reported they benefitted from developing relationships with peers participating in FCD and included friendship and camaraderie; sharing and learning from others with similar experiences; and reinforcing messages on adherence, retention and general health.

## Discussion

The findings of this study suggest that the provision of HIV care to paediatric and adolescent patients through an FCD can play an important role to increase adherence to clinic appointment schedule. We found evidence that paediatric and adolescent patients in FCD facilities were more likely to be adherent to their appointment schedule compared to those in non-FCD facilities, thereby having the opportunity to be adherent to ART.

Further, qualitative interviews suggested an improved quality of ART services through health education and increased psychosocial support. This finding is supported by qualitative evidence from another Ugandan study reporting that among HIV positive adolescents, stigma, self-image, identity and peer-pressure were identified as some of the main barriers preventing adherence.[19] Evidence from this study provides some evidence to support an innovative approach to improve low adherence rates in light of the need for better age-specific strategies to improve low adherence rates among paediatric and adolescents.[20, 21]

The study observed no significant improvement in paediatric and adolescent retention, which may be due to the very high retention outcome found among study facilities and the limited follow-up period. The power to detect an 8% effect size difference between study arms was based on a conservative assumption of 70% retention based on MOH data regarding retention levels among 12-month initiate patients. Actual retention rates at greater than 90% observed in this study were higher than expected, and therefore it would likely not have been feasible to detect any further significant improvement in retention.

Furthermore the MOH retention definition involved a 90-day time period that was likely too broad within this study’s context to detect any significant difference in retention. However thanks to the shorter 30-day interval associated with appointment scheduling, any such delays in ART clinic attendance may have been more easily detected. Therefore while a client may be classified as non-adherent to their appointment schedule, they may nevertheless be retained in care if ever attending within the retention outcome 90-day timeframe; our adherence definition provided a more sensitive measure of a client’s clinic appointment behavior. This may explain why significant differences in adherence outcomes were detected without an apparent effect on retention.

Our findings are consistent with those from a Nigerian study, where a paediatric ART program involving children and their caregivers found that a Kiddies’ Club intervention, mainly involving psychosocial and community support, improved child adherence to ART.[22] More generally, adherence clubs among ART patients have improved adherence rates through strong community and psychosocial support.[23] These results suggest that the benefits of FCD may be most apparent through encouraging patients to closely adhere to their appointment schedule, thereby improving their access to a continuous supply of ARVs.

Qualitative findings indicated participant reactions and feedback on the FCD program were largely positive. Participants suggested that a positive impact was experienced in strengthened health education, reduced clinic congestion and visit length, improved facility and appointment management, enhanced family and peer social support. No data were collected in control sites about patient satisfaction with status quo programming, but these results do provide support for understanding the mechanisms of positive changes in adherence that were observed. Specifically, health education seems to have helped patients understand why adherence is important, and support from family and peers may have helped patients to follow through on attending appointments according to the schedule.

This study is likely not directly generalizable to all other settings as the local Ugandan context may play an important role in the need, implementation and experience with the FCD intervention. Differences regarding the local background and context of Ugandan patients attending ART clinics, and the healthcare workers providing ART services, could lead to important differences that prevent the generalizable application of results from this study.

Additionally, there may have been selection bias towards the FCD group caused by differential data quality issues occurring between study arms. As part of the FCD intervention, participants were clearly identifiable by color-coded folders while the control arm provided the standard of ART care that did not use such a method. Therefore if control site participants whose ART files were not as well managed also happened to be more likely sick, non-adherent to ARV treatments, and less likely to attend their appointment schedule, these individuals would therefore be differentially represented across study arms. This selection bias would lead to an under-estimate of the true FCD effect size.

Finally, it is important to consider further potential bias which may have played a small role on the study’s internal validity, as the quality of the FCD intervention was dependent on a variety of factors including the implementing partner, ART clinic facility operations, and the delivery of services by healthcare workers. Any variability of aforementioned factors across each respective facility in the study would potentially lead to misclassification of exposure. Differential misclassification may also have occurred due to possible inconsistencies in how facilities implemented the intervention, and as such this would have led to an underestimated effect size.

### Limitations

The results of this evaluation should be interpreted in light of several limitations. First, the evaluation measured outcomes after six months of intervention, because the MOH was interested in rapid evidence to inform policy. It was therefore not possible to know whether additional improvements would be achieved during a longer period or whether the positive changes that we did observe in adherence would have been maintained. Therefore the short study period may have contributed to the high levels of retention seen across both study arms, which in turn may mask any potential benefit of the intervention on retention outcomes. Sensitivity analyses investigated model estimate robustness according to sampling strata, sub-population analysis focusing on new ART initiates, and finally using Cox-proportional hazards models accounting for follow-up time. These all confirmed our conclusion that patients in the intervention arm were no more likely to be retained than those in the control arm.

Second, data collection was conducted for all patients ever active on ART at any point during the study period including patients initiating at first visit. Therefore, we were not able to measure retention and adherence of 6-month initiates, as sub-population analyses using patient groups in care for less than 12 months were underpowered.

Third, it was beyond the scope of this project to measure adherence with biomarkers, so we relied on assumptions about adherence to appointments and the opportunity to be adherent to ARV tablets, but not actual adherence to ARV tablets.

Fourth, a large amount of missing data (n = 1359) resulted from an inability to find the ART care cards of patients who were otherwise listed as clients in facility ART registers. These missing ART care cards meant we had no data on clients who may have otherwise been eligible for inclusion in the analysis. However due to significant efforts led by mentors to ensure data quality and completeness across all study facilities, we are confident that any missing data is equally likely among adherent and non-adherent patients across control and intervention arms, therefore any misclassification would be non-differential, and hence the measure of association would be unaffected.

Fifth, operational feasibility meant that this study was conducted in partner-supported sites within 3 regions of Uganda. While this may therefore limit generalizability of findings to non-partner supported sites, this group nevertheless accounts for only approximately 10% of ART sites in Uganda. Therefore we believe the findings of this study are thus applicable to a majority of facilities where patients seek HIV care.

Finally, the verification of FCD-ineligible patients presented a challenge. The files of FCD eligible children were visibly color-coordinated to differentiate them from other patient files, however their family members did not benefit of the same system. Consequently, confirming whether an adult patient was linked with an FCD child was difficult to conclude, and despite the fact that roughly 90% of clinic patients were adults, it was not possible to determine whether they were definitively linked with an FCD child. Therefore due to our inability to confirm the link between any adult and an FCD child, our secondary outcome analyses used the general adult population without final confirmation of their potential link to the FCD intervention.

In the qualitative component of the assessment, the views of participants interviewed may not be fully representative of the patients and health care workers in the study or in Uganda more generally. This may be particularly true in the case of adolescents, where we faced challenges in recruiting the expected number participants of the appropriate age. Many adolescents were found to be away at boarding school and these results are unlikely to capture their views. Additionally, some adolescent groups were too small for the group to engage in consensus building and disagreements for which focus groups are valuable. However, we sought to visit a wide range of types of facilities and to recruit participants with varied positions and backgrounds. It is also possible that some degree of bias may have been introduced as a result of using facility staff to recruit adolescents and caregivers. This qualitative recruitment strategy was used due to feasibility constraints associated with conducting multiple visits to the facility.

### Conclusion

This evaluation utilized the standard techniques in implementing a new program within the MOH of Uganda. As such, we provide real-world findings; at 6-months following the intervention the FCD intervention improved paediatric and adolescent adherence to appointment schedules, and did not have a negative impact on the adult ineligible population at the same facilities. We did not find evidence to support the efficacy of FCD to improve patient retention in care at 6 months. Qualitative findings suggest that improvements were supported through the mechanisms of increased knowledge based on health education lessons and psychosocial support. Since further research into the impact of the FCD on sub-populations (such as those in ART care for less than 12 months) would require a large, potentially unfeasible study, this could be monitored during routine assessments of retention in cohorts.

## Supporting information

S1 Table CONSORT checklist for randomised trials. (DOC)

S1 Dataset Spreadsheet containing paediatric and adolescent level child data. (CSV)

S2 Dataset Spreadsheet containing adult level data. (CSV)
